# Near Infrared Photoimmunotherapy for Locoregionally Recurrent Oropharyngeal Squamous Cell Carcinoma at Tongue Base After Chemoradiotherapy: A Case Report and Literature Review

**DOI:** 10.7759/cureus.74742

**Published:** 2024-11-29

**Authors:** Shunji Tamagawa, Katsuya Okuda, Daisuke Nishikawa, Masamitsu Kono, Muneki Hotomi

**Affiliations:** 1 Department of Otorhinolaryngology-Head and Neck Surgery, Wakayama Medical University, Wakayama, JPN; 2 Department of Head and Neck Surgery, Aichi Cancer Center, Nagoya, JPN

**Keywords:** airway management, control perioperative pain, laryngeal edema, near infrared photoimmunotherapy, oropharyngeal carcinoma

## Abstract

Near-infrared photoimmunotherapy (NIR-PIT) is a newly developed treatment. We report a successful case of NIR-PIT for post-irradiation locoregionally recurrent oropharyngeal cancer at the tongue base.

A 60-year-old man following primary treatment for oropharyngeal cancer at the tongue base by endoscopy (rT1N0M0). He had been treated by concurrent chemoradiotherapy. NIR-PIT was performed under general anesthesia at the tongue base. Successful therapeutic effects were obtained with no postoperative functional impairment.

This case report highlights two important management issues: tracheostomy and pain management after NIR-PIT. NIR-PIT for head and neck cancer will provide a curative treatment option for the locoregional recurrent or unresectable disease following radiotherapy and surgery. It was considered important to ensure sufficient optical tissue penetration range, control pain in the perioperative period, and take measures to prevent edema in the airway.

## Introduction

Oropharyngeal cancer is a type of head and neck cancer. Although recent increases in human papillomavirus-associated oropharyngeal cancer show better responses for chemoradiation therapy, the recurrence cases of oropharyngeal cancer still show a poor prognosis. Because the base of the tongue plays an essential role in the phonation and the swallowing process, surgical resection of oropharyngeal cancer tends to be avoided, and chemoradiotherapy is widely applied for treatment. However, the additional radiation therapy after primary radiation for oropharyngeal cancer is difficult to achieve. Salvage surgery has been the alternative treatment option. In contrast to the difficulties in the salvage treatment of oropharyngeal cancer, the recurrence happens in 30-40% of stage III/IV oropharyngeal cancer cases, even after the primary chemoradiation. It is also estimated that salvage surgery is successful in only 46% of cases [1]. The main reasons for the difficulty of salvage surgery are not only the local condition of the tumor but also the poor general condition. The ideal salvage surgery is therefore one that allows a more reliable surgical resection and is minimally invasive to the overall condition. Approaches include pharyngeal incision through an external cervical incision, transoral tumor resection with the aid of an endoscope, and in recent years, transoral robotic surgery has been attempted [2]. However, salvage surgery after chemoradiation and/or surgery carries higher risks of life-threatening complications such as wound dehiscence and carotid artery rupture caused by delayed wound healing [1]. The salvage surgery also strongly reduces swallowing function. Therefore, treatments with chemotherapy, molecular-targeted medicine, and immune checkpoint inhibitors are frequently performed for recurrent cases. However, these treatments have not provided satisfactory results [3].

The three mainstream cancer treatments in clinical practice have been surgery, radiation, and chemotherapy. However, they have not achieved the ability to attack, kill, and remove only cancer cells, while all of these treatment modalities are effective in reducing the number of cancer cells. As a result, patients suffer from post-treatment complications, side effects of treatments, and weakened immunity. A novel treatment of near-infrared photoimmunotherapy (NIR-PIT) is highly selective against cancer cells and provides different approaches to cancer treatments [4]. NIR-PIT is a newly developed treatment combining the photosensitizer, sarotalocan sodium IRdye700DX, with the chimeric anti-human epidermal growth factor receptor (EGFR) monoclonal antibody, cetuximab. The first phase I/IIa trial (RM-1929-101) for patients with unresectable head and neck SCC was completed in 2017 with a global phase III clinical trial (RM-1929-102) from 2019 to 2024 [5]. The Japanese Ministry of Health, Labour, and Welfare has approved NIR-PIT (Alluminox TM) for unresectable locally advanced or unresectable locoregionally recurrent head and neck cancers in 2021. However, there are still a few case reports about this novel treatment [6-19]. It is important to accumulate the cases of NIR-PIT.

Here, we report a successful case of NIR-PIT for locoregionally recurrent oropharyngeal cancer at the tongue base after chemoradiation. Successful therapeutic effects were obtained with no postoperative functional impairment.

## Case presentation

**Figure 1:**
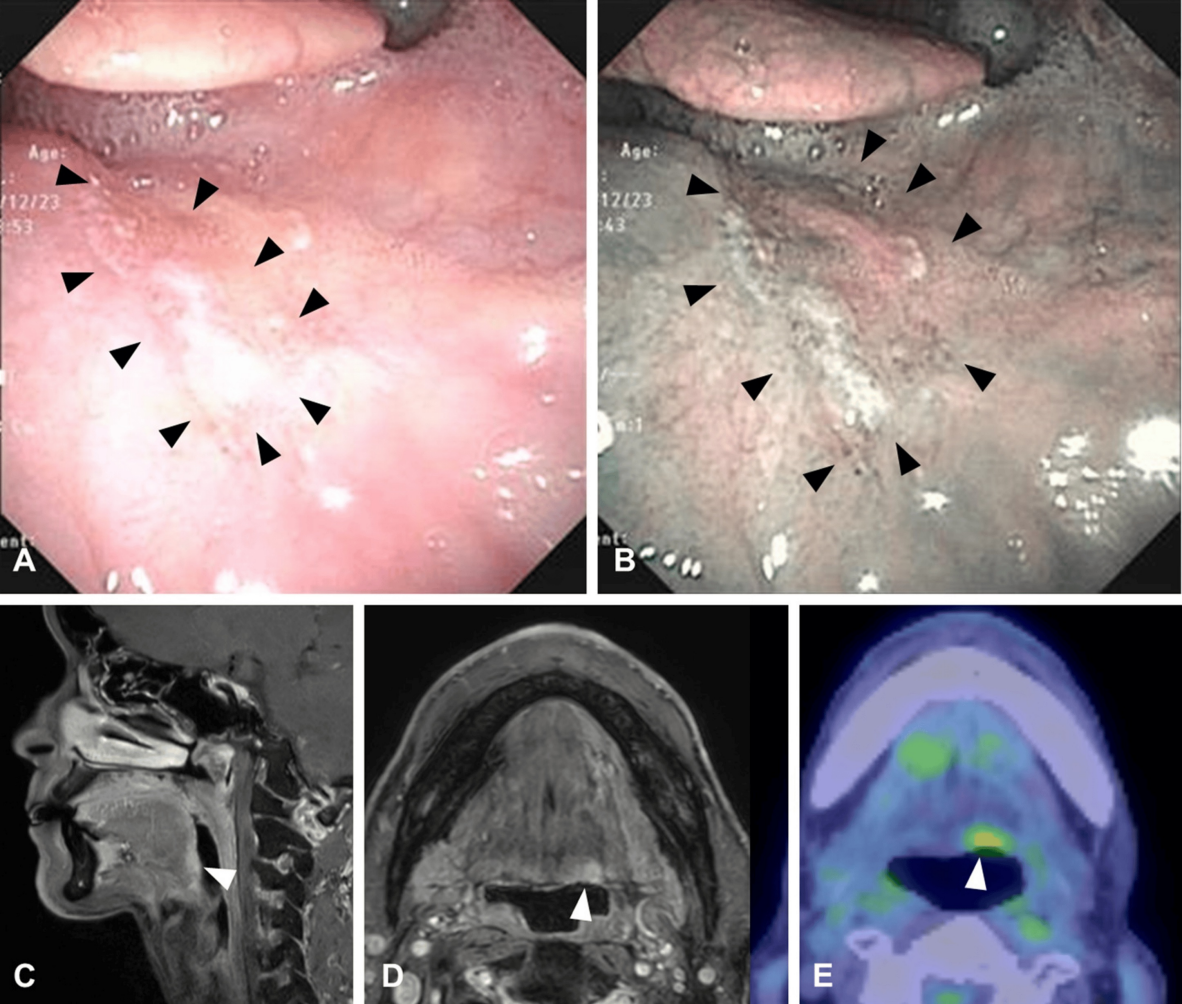
Locoregional findings. A. laryngeal endoscope under normal light. A tumor with an irregular surface approximately 15 mm in size was observed on the left side of the base of the tongue. B. laryngeal endoscope under narrowband optical endoscopy. The surface of the tumor is keratinized, and irregular blood vessels are observed in the surrounding area. C. and D. Gd-enhanced MRI. A contrast-enhanced tumor approximately 1 cm in size was observed at the base of the tongue, and invasion approximately 1 cm deep was suspected. E. PET-CT. FDP accumulation at SUV=3.98 was observed in the same area as contrasted by MRI.

A 60-year-old man presented with an endoscopic mass lesion at the base of the tongue (Figure 1A). He had a history of concurrent chemoradiotherapy for oropharyngeal cancer (stage unknown) twelve years ago. During follow-up, endoscopy revealed a locoregional recurrence of oropharyngeal cancer at the tongue base (p16-negative SCC, rT1N0M0). In addition, he had a history of right partial glossectomy for right lingual cancer (SCC, cT1N0M0) seven years ago and transoral resection of left buccal mucosa cancer (SCC, cT1N0M0) five years ago. He had a 40-year history of alcohol consumption and a 25-year history of smoking.

Narrowband optical endoscopy revealed a B1 blood vessel approximately 20 x 20 mm in size. The lesion extended from the midline of the tongue base to the vallecula (Figure 1B). Contrast-enhanced MRI showed a 10 x 10 mm lesion with contrast enhancement and high intensity at T1, consistent with the left tongue base lesion confirmed by endoscopy (Figures 1C, 1D). PET-CT showed FDP accumulation at SUV=3.98 in the same site. However, no metastatic lesions were found (Figure 1E). The pathological examination revealed squamous cell carcinoma and EGFR expression was not evaluated. The recurrence occurred within the radiation field of previous concurrent chemoradiotherapy, and the patient had undergone multiple oral resections for tongue and buccal mucosal cancer. NIR-PIT was chosen because the case had risks of delayed wound healing and severe dysphagia following additional oral surgical resections.

Photoimmunotherapy was performed under general anesthesia for the locoregional recurrence of oropharyngeal cancer at the tongue base (p16-negative squamous cell carcinoma rT1N0M0). Briefly, cetuximab sarotalocan sodium was administered intravenously at the standard dose of 640mg/m2. Then, he was managed in a dark room with an illumination under 120 lux to avoid photosensitivity.

A tracheostomy was performed under general anesthesia to secure the upper airway. In addition, 100 mg of hydrocortisone sodium succinate was intravenously administered to reduce laryngeal edema during surgery. 

**Figure 2:**
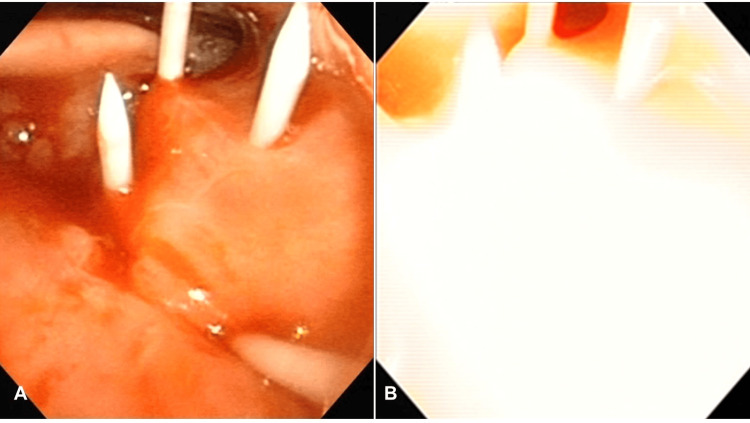
NIR-PIT A. Laryngeal endoscope during surgery. Four cylindrical diffusers were inserted at 5 mm intervals. B. Laryngeal endoscope during photoimmunotherapy.

Laser illumination using a photodynamic therapy semiconductor laser and probes (BioBrade laser and BioBrade frontal diffuser, Rakuten Medical, Tokyo, Japan) was performed for 5 minutes, 24 hours after cetuximab sarotalocan sodium administration. A 2-3 mm safety margin from the tumor was designed. After confirmation of the lesion by bimanual examination, a test puncture was performed from the neck using a 22 Gy needle under ultrasound guidance, avoiding the lingual artery. After re-confirmation of the puncture direction, the first puncture was made, and then the second puncture was made parallel to the first one (Figures 2A, 2B).

**Figure 3:**
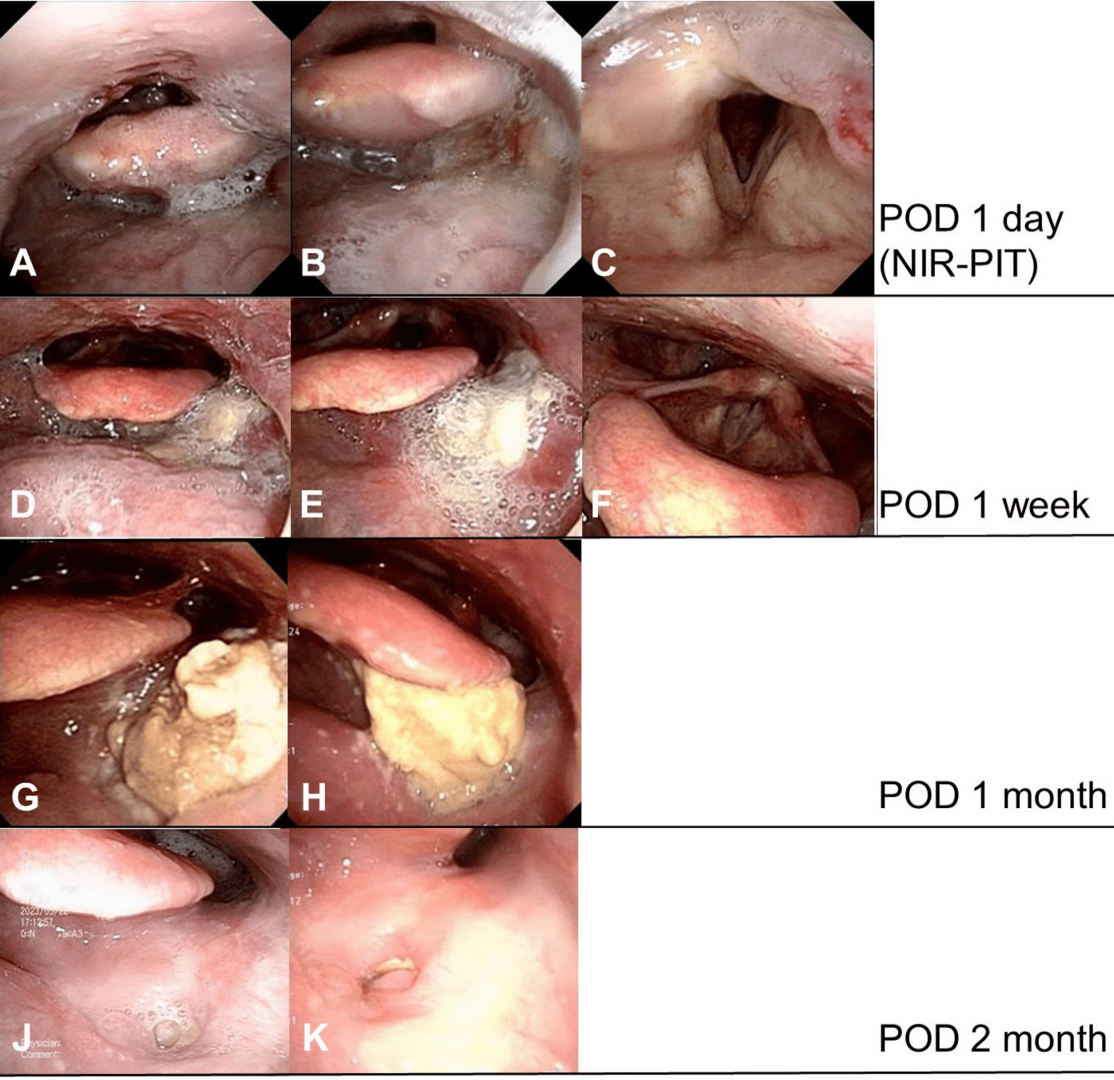
Clinical course of locoregional recurrence after NIR-PIT. A, B, and C. laryngeal endoscope after 24 hours. There was significant edema at the base of the tongue, and the edema had spread to the tongue base of the epiglottis. The glottis is patent, and edema has spread along the left aryepiglottic fold to the arytenoid region. D, E, and F. laryngeal endoscope after one week. The edema of the epiglottis has improved, and the epithelium at the base of the tongue has become necrotic and has begun to fall off. G and H. laryngeal endoscope after 1 month. Epithelial shedding progresses, and necrotic tissue accumulates. J and K. laryngeal endoscope after 2 months. Epithelial necrosis is sloughed off and scarring progresses.

A day after photoimmunotherapy, severe mucosal edema appeared from the epiglottis to the tongue base mucosa (Figures 3A-3C).

The patient stayed in a dark room under 120 lux of light to avoid photosensitivity for six days after photoimmunotherapy. He was finally discharged seven days after NIR-PIT. There were no complications, including wound pain or laryngeal edema. It was recommended to avoid exposure of skin and eyes to sunlight directly for four weeks. One week after surgery, the mucosal epithelium gradually fell off (Figures 3D-3F), and the mucosa and submucosa around the tumor became necrotic one month after surgery (Figures 3G, 3H). The irradiated area became a scar two months after surgery (Figures 3J, 3K). A biopsy revealed no malignant findings. Ten months after therapy, he had no recurrence or metastasis.

## Discussion

The treatment for locoregionally recurrent oropharyngeal carcinoma is a clinical dilemma. This case report presents the therapeutic efficacy of NIR-PIT with accurate clinical findings. This case illustrates the importance of airway and pain management after NIR-PIT [6,8,13,14,17].

The indication of NIR-PIT remains controversial. The most common site is reported to be the oropharynx [12]. In order to understand the benefits of NIR-PIT, it is of great value to report clinical cases. EGFR expression was not evaluated in the current case. The active evaluation is considered to contribute to the accumulation of evidence, as EGFR expression can be a possible predictor of the efficacy of this treatment [12,20]. The advantage of NIR-PIT is that their antitumor effects are in the tumor periphery without damaging normal tissues surrounding tumor cells [4,11]. However, in clinical practice, it is considered sufficient to treat head and neck squamous cell carcinoma without confirming the intensity of EGFR expression, as EGFR is highly expressed in head and neck squamous cell carcinoma. The range of light transmission within tissue using a cylindrical diffuser is approximately 10 mm in radius. Thus, the lesion of cancer should be within the range of infrared illumination. During treatment, care must be taken to avoid tumors near large blood vessels, fistula formation due to tissue defects after treatment, and laryngeal edema. In this case, four cylindrical diffusers were inserted at 5 mm intervals through the neck surface under a laryngeal endoscope and ultrasound guidance. The lingual artery was confirmed under ultrasound guidance to avoid vascular damage. The postoperative tissue necrosis occurred at 20 to 30 mm after NIR-PIT. Pharyngeal fistula is one of the major complications of NIR-PIT. The tumor, in this case, was located in a shallow layer. Mucosal necrosis did not develop in the deeper tissues and fistula.

Laryngeal edema is a life-threatening side effect of NIR-PIT and is associated with the lesions targeting NIR-PIT adjacent to the larynx [6,13]. Its occurrence will be based on NIR-PIT inducing tumor cell necrosis by singlet oxygen and reactive oxygen species and releasing damage-associated molecular patterns (DAMPs), which stimulate inflammatory cytokine production [4]. The subsequent increase in vascular permeability will contribute to induced edema [4]. Administration of hydrocortisone sodium succinate during operation is recommended to reduce postoperative laryngeal edema. Whether prophylactic tracheostomy should be universally applied for all patients undergoing PIT remains controversial. NIR-PIT produces locoregional reactive oxygen, which causes edema. Nishikawa et al. reported the two cases of oropharyngeal cancer cases performing prophylactic tracheotomy [11]. In contrast, Okamoto et al. reported that emergency tracheotomy was required after NIR-PIT in two cases of oropharyngeal cancer [6]. However, Kushihasi et al. reported that emergency tracheotomy was required even after nasopharyngeal treatment [16]. The venous return route may be altered after reconstructive surgery or radiation therapy. A prophylactic tracheostomy would be required if NIR-PIT is performed close to the larynx. The oral cavity and oropharynx are close to the larynx and provide venous reflux to the larynx. On the other hand, laryngeal edema has not been reported with NIR-PIT for cutaneous cancers or parotid cancers. Strong mucosal edema was observed from the epiglottis base surface to the left tongue base tonsil on the day after surgery, even distant from the larynx in our case. Airway emergencies can be life-threatening and should be diligently monitored as a potential complication of NIR-PIT. It is better to consider the implementation of prophylactic tracheostomy as a standard procedure for all patients given the inherent risks associated with this treatment approach [6].

One of the specific procedures of this treatment is to keep the patient in a dark environment for a long time. We believe that psychological assessment for patients is also important in the implementation of photoimmunotherapy. Additionally, supportive therapy for pain is particularly important because NIR-PIT for the head and neck is a tissue-destructive treatment [11,14]. In clinical trials, approximately 12% of patients experienced Grade 3 or higher pain at the treatment target site [5]. Fentanyl citrate was administered via the PCA pump to reduce pain. In addition, oral acetaminophen was administered after the NIR-PIT. The NRS (Numerical Rating Scale) was controllable at 2-5, so good results were achieved. However, it is necessary to continue pain control for approximately 5 weeks after NIR-PIT. Okamoto et al. reported that NIR-PIT did not significantly change functional scales and global health status as well as domain scales [7]. NIR-PIT may contribute to the maintenance of QOL after treatment. The literature review is shown in Table 1.

**Table 1:** Literature review.

Authors and reference number	Number of cases	Target site	Tracheostomy	pain control	Summary
Cognetti DM. et al. 2021 [5]	30	Head and neck squamous cell carcinoma (except Nasopharyngeal carcinoma)	One out of 30 cases	Unknown	In one patient, tissue swelling resulted in airway obstruction which required temporary tracheostomy.
Okamoto I. et al. 2024 [6]	2	Oropharynx	Two cases (emergent tracheostomy)	Unknown	One patient showed respiratory distress three hours after the end of the operation, while another patient developed respiratory distress in the hospital room six hours after surgery.
Okamoto I. et al. 2022 [7]	9	Three cases oropharynx,2 cases reconstructed skin valve/oropharynx, 2 cases oral cavity, 1 case cervical lymph node,1 case maxillary sinus	Laryngeal edema GradeⅡ,2 cases	GradeⅠ,１ case GradeⅡ,7 cases GradeⅢ,1 case	Edema of the larynx was observed in three patients. But, no patient required emergency tracheostomy.
Okamoto I. et al. 2022 [8]	1	Neck skin	After total laryngectomy	NSAIDs alone	Nonsteroidal anti-inflammatory drugs were administered to relieve the pain associated with the adverse reaction.
Nishimura M. et.al. 2024 [10]	1	Nasopharynx	Unknown	Unknown	Lemierre's syndrome that developed after HN-PIT for recurrent nasopharyngeal carcinoma.
Nishikawa D. et.al. 2022 [11]	2	Oropharynx	Prophylactic tracheostomy	Intravenous administration of fentanyl, NSAIDs and acetaminophen	Continuous intravenous fentanyl was administered intraoperatively for prophylaxis of postoperative pain.
Idogawa H. et al. 2023 [14]	1	Nasopharynx	No (Grade II laryngeal edema)	Grade II	Grade 2 adverse events have occurred (pain, laryngeal edema, suspected osteomyelitis)
Koyama S. et al. 2023 [15]	1	Maxillary sinus	Unknown	Unknown	Cancer pain was reduced after NIR-PIT.
Kushihashi Y. et al. 2022 [16]	1	Nasopharynx	Emergent tracheostomy	Analgesics	On the first postoperative day, emergency tracheostomy was performed due to upper air way edema and difficulty with oral intubation.
Omura G. et al. 2023 [17]	1	Nasopharynx	No laryngeal edema	No pain	There were no complications, including wound pain or laryngeal edema.
Kishikawa T. et al. 2023 [18]	1	Oropharyngeal carcinoma (subcutaneous recurrence)	Unknown	Unknown	The patient had no serious adverse events.
Makino T. et al. 2024 [20]	1	Parotid gland	Unknown	Unknown	The patient experienced pain a few days after every cycle of the treatment.

## Conclusions

We encountered locoregionally recurrent oropharyngeal squamous cell carcinoma. Postoperative laryngeal edema was observed, but safe airway management was achieved by performing a prophylactic tracheostomy. Additionally, sufficient pain management was achieved using a postoperative fentanyl citrate PCA pump and oral acetaminophen. NIR-PIT will provide a curative treatment option for locoregional recurrent oropharyngeal cancer after chemoradiotherapy and surgery. It was considered important to ensure sufficient optical tissue penetration, control perioperative pain, and take measures to prevent airway edema.
